# Supplementary material for: Complexome profiling of the Chlamydomonas psb28 mutant reveals TEF5 as an early PSII assembly factor
Source: Plant Cell. 2025 Mar 17;37(6):koaf055. doi: 10.1093/plcell/koaf055 (PMC12164590; doi:10.1093/plcell/koaf055)
Supplement: koaf055_Supplementary_Data [file koaf055_supplementary_data.zip › psb28_interactive.zip!/plots/intensity/qualityPlots/peptides/CAM14_Cre10.g464600.t1.1.html]

### Cre10.g464600.t1.1

Trivial name: CAM14  
SeqCoverage [median]: 18.70  
Mass: 21.16 kDa  
  
 

| Sequence | MissedCleavage | Mass [Da] | wtIntensity | muIntensity | Score |
| --- | --- | --- | --- | --- | --- |
| AELAAGLGDLGR | 0 | 1141.61 | 733.79 | 1177.02 | 74.94 |
| GMALELGVDER | 0 | 1188.58 | 629.39 | 448.55 | 120.99 |
| LNAAEAQR | 0 | 871.45 | 828.28 | 0.00 | 137.90 |
| NADEVLR | 0 | 815.41 | 374.05 | 1618.35 | 124.59 |
|  |  |  | 2565.5 | 3243.9 |  |
